# Mazur’s Peer Instruction in Medical Education: A Systematic Review and Meta-Analysis

**DOI:** 10.1007/s40670-026-02744-1

**Published:** 2026-04-28

**Authors:** Mohammad Aldalou, Asa Kizer, Jamison C. Hanley, Ahmad R. Hakim, Jill Deaver, Adam B. Wilson, William S. Brooks

**Affiliations:** 1 Department of Medical Education, Marnix E. Heersink School of Medicine, University of Alabama at Birmingham, Birmingham, AL USA; 2 Department of Clinical, Academic, and Research Engagement, Lister Hill Library of the Health Sciences, University of Alabama at Birmingham, Birmingham, AL USA; 3 Department of Anatomy and Cell Biology, Rush University, Chicago, IL USA

**Keywords:** Peer instruction, Active learning, Learning outcomes, Medical education, Health professions education

## Abstract

Peer Instruction (PI) has demonstrated benefits in STEM education, but its effectiveness as a distinct instructional method in health professions education remains uncertain. This systematic review identified 17 eligible studies implementing Mazur’s PI framework in medical and dental education, with 12 contributing to meta-analyses. PI yielded large within-session conceptual gains (Hedges’ g ≥ 1.48; *p* < 0.001) but showed no performance superiority over alternative instructional approaches (*p* = 0.082). 74% of learners reported PI to be helpful (*p* < 0.001). Evidence for long-term retention or transfer remains limited. Curricular adoption should be guided by logistical feasibility and learning objective alignment rather than expectations of superior performance over other established active strategies.

**Supplementary Information:**

The online version contains supplementary material available at 10.1007/s40670-026-02744-1.

## Introduction

Active instructional approaches are increasingly emphasized in higher education as alternatives to traditional lecture-based instruction, as evidence suggests that lectures alone may be less effective at promoting conceptual understanding and higher-order reasoning [1–10]. In medical and dental education, instruction has similarly shifted, albeit more gradually, away from faculty-centered, lecture-heavy teaching toward student-centered pedagogies intended to support durable learning and knowledge transfer required for future practice [8, 11–14].

Within this broader shift, active learning is neither a single nor uniform intervention. Instructional approaches commonly classified as active learning, such as case-based learning (CBL), team-based learning (TBL), and problem-based learning (PBL), each well-established in health professions education, differ considerably in their goals, structures, instructional sequences, and educational contexts [8, 13, 15–19]. Additional active instructional strategies have been introduced in health professions education less consistently, and the evidence supporting their use is often dispersed across studies and settings. This makes it difficult to interpret findings or to meaningfully compare approaches, contributing to uncertainty regarding which strategies warrant broader adoption within already crowded curricula.

**Fig. 1:**
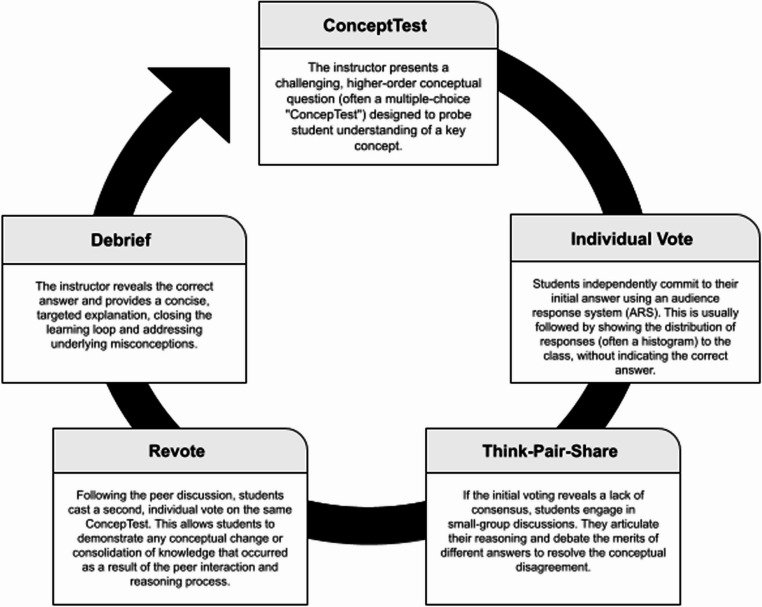
The peer instruction cycle illustrating the structured sequence of Mazur’s method

Peer Instruction (PI), developed by Eric Mazur in the 1990s to address students’ poor conceptual understanding in introductory physics, is one such active instructional method. A typical PI cycle includes five steps (Fig. 1): (1) an instructor poses a conceptual question (e.g., a multiple-choice “ConcepTest”), (2) students commit to an individual answer using an audience response system (ARS), (3) students discuss their reasoning in small peer groups, commonly following a think–pair–share format, (4) students submit revised answers through the ARS, and (5) instructors review response data and explain the correct answer [3, 20–23]. ConcepTests are short conceptual questions, usually multiple choice, designed to probe the students’ understanding of core principles rather than recall of isolated facts, with answer choices that typically reflect common misconceptions [20]. In some implementations, the PI cycle is preceded by preparatory activities (e.g., assigned readings, pre-session questions, or recorded lectures) intended to establish foundational knowledge prior to engagement.

The PI cycle requires learners to commit to an initial answer, explain their reasoning, and then revise their thinking through peer discussion and feedback, a sequence that supports conceptual change and deeper understanding. This approach draws on complementary learning perspectives. First, from a cognitive constructivist view, committing to an answer and confronting discrepancies forces active restructuring of prior knowledge rather than passive reception. Second, from a social constructivist view, learning is mediated through dialogue, as students explain, defend, and negotiate ideas with peers. Third, PI incorporates elements of productive failure, as students first attempt to solve a challenging conceptual problem on their own, often exposing incomplete or incorrect reasoning, and this initial struggle prepares them to learn more deeply from subsequent peer discussion and instructor clarification [22, 24–28].

Over the past two decades, Mazur’s PI method has demonstrated efficacy in science and engineering education. In undergraduate physics courses, replacing or supplementing traditional lectures with PI has been associated with improvements in students’ conceptual understanding and problem-solving performance [25]. For example, Crouch and Mazur’s 10-year study reported that classes taught with PI were associated with statistically significant improvements in both conceptual understanding and quantitative problem-solving performance, including higher performance on quantitative exam items across cohorts (*p* = 0.001, effect size = 0.34-0.57) [3]. Similar findings have been replicated across multiple STEM disciplines and institutions, suggesting that PI may have relevance beyond its original disciplinary context [1, 22, 24].

The adoption of PI in health professions education has lagged behind other STEM fields [29]. Within medical and dental curricula, PI remains underexamined and is often conflated with broader peer-based or collaborative learning approaches [30]. Although initial studies exist, reported outcomes vary across studies and settings, and the literature has not been consolidated in a manner that allows clear interpretation of PI’s educational impact as a defined instructional method within these fields [30–33].

This presents a compelling question: Does Peer Instruction produce the same advantages in undergraduate and graduate medical and dental education as has been observed in other STEM educational settings? To date, no study has quantitatively synthesized the available evidence on PI in these fields. The purpose of this study was therefore to conduct a systematic review and meta-analysis of PI in undergraduate and graduate medical and dental education to evaluate its effectiveness as a distinct instructional method and to inform evidence-based curricular decision-making.

## Methods

### Reporting Framework

This systematic review and meta-analysis was conducted in accordance with the Preferred Reporting Items for Systematic Reviews and Meta-Analyses (PRISMA) 2020 guidelines [34]. Ethical approval was not required, as this study synthesized data from previously published literature.

### Search Strategy

A literature search of five electronic databases: PubMed, CINAHL, Scopus, Web of Science, and Embase, was conducted in September 2024 by a medical librarian (J.D.) experienced in health professions education reviews. The search was specifically designed to capture studies that follow Mazur’s PI framework, which is characterized by conceptual questioning, peer discussion, and instructor-led discussion or feedback. This specificity was necessary to distinguish PI from broader “peer learning”, “peer teaching,” or “peer tutoring” terms often used interchangeably in the literature. Each search combined controlled vocabulary and free-text terms related to medical education, dental education, and peer instruction. The systematic search strategy is provided in Appendix A.

No restrictions were placed on publication year. Searches were limited to English-language, peer-reviewed journal articles involving human participants. Only primary empirical studies were eligible for inclusion; editorials, commentaries, narrative or systematic reviews, conference proceedings, and dissertations were excluded. Reference lists of all included articles and relevant reviews were manually screened to identify additional eligible studies not retrieved through database searching.

### Study Selection

Study eligibility was defined using a Population–Intervention–Outcome (PIO) framework. Eligible studies involved undergraduate or graduate trainees in medical or dental education; studies involving students from other health professions (e.g., nursing, veterinary medicine) or non-health disciplines were excluded. Regarding the intervention, eligible studies must have implemented Mazur’s PI framework or its core elements (conceptual questioning, peer discussion, instructor feedback), recognizing that implementation may vary across educational contexts. Studies describing general peer teaching, peer tutoring, peer-assisted learning (PAL), or collaborative group work that lacked this structured sequence were excluded. For inclusion in the meta-analysis, studies were required to report quantitative outcomes [35] related to academic performance (e.g., exam scores), knowledge gain, or learner perceptions. Consistent with a Population–Intervention–Outcome (PIO) framework, comparator eligibility was intentionally defined broadly to reflect prevailing practices in the medical education literature, where instructional studies commonly employ heterogeneous designs and diverse comparator conditions. Accordingly, eligibility was not limited to comparisons with passive lecture formats; instead, both comparative and within-group study designs were included to permit evaluation relative to the range of instructional approaches reported in the literature.

All identified records were imported into Covidence [36] (Veritas Health Innovation, Melbourne, Australia) for screening and workflow management. Five reviewers independently screened 334 titles and abstracts against predefined inclusion and exclusion criteria. Two reviewers screened each record, and a third reviewer resolved discrepancies. Full-text screening was conducted independently by two reviewers, demonstrating 94.9% agreement (Cohen’s κ = 0.89), with disagreements resolved through discussion and consensus [37].

### Data Extraction

Data extraction was performed by one reviewer (M.A.) and verified by a second reviewer (W.S.B.) using a standardized form developed through iterative testing and revision. Extracted variables included: study identification (authors, year, citation) and educational context (country, institution, learner level, discipline), study duration, design type (e.g., quasi-experimental posttest-only with control, quasi-experimental pre–post design, pre–post design with control, randomized controlled designs, and historical cohort designs), sample size, intervention components, control condition (if applicable), assessed outcomes (performance, confidence, satisfaction, conceptual gain), measurement instruments (e.g., multiple-choice questions, Likert-scale surveys), and reported quantitative data (means, standard deviations, and p-values).

When key data were unavailable, the corresponding authors were contacted via email to obtain the missing data. For studies reporting ranges rather than standard deviations that could not be verified due to the authors’ unresponsiveness, standard deviations were estimated using the formula established by Wan et al. (2014) [38].

### Quality Assessment

The quality of all included quantitative studies was assessed using the Medical Education Research Study Quality Instrument (MERSQI), which evaluates six domains: study design, sampling, type of data, validity evidence, data analysis, and outcomes [39]. Each domain consists of specific criteria, with scores ranging from 0 to 3 or 1 to 3, depending on the category. A study can obtain a maximum score of 18, with higher scores denoting higher quality. Quality appraisal was completed by one reviewer (M.A.) and independently verified by a second reviewer (W.S.B.). No studies were excluded based on the MERSQI score. Given the limited number of eligible studies and the dominance of quasi-experimental designs in medical education research, applying a strict quality cutoff risked excluding informative studies that contribute meaningful context-specific insights. MERSQI scores were therefore used descriptively to characterize the overall strength of the evidence base rather than as a criterion for study exclusion.

### Data Analysis

Data management and cleaning were performed in Microsoft Excel (version 2306, Microsoft Corporation, Redmond, WA). Study characteristics were summarized using descriptive statistics. Outcome measures were categorized into three domains: (1) within-session conceptual gains, defined as the change in the proportion of correct responses from pre- to post-peer discussion within a single PI session; (2) learning outcomes, defined as performance on assessments administered at the end of a session, course block, or semester; and (3) learner perceptions, defined as self-reported evaluations of PI’s helpfulness, including perceived support for learning, reasoning, and content understanding.

Meta-analyses were conducted using Comprehensive Meta-Analysis (version 4, Biostat, Englewood, NJ) [40]. Given the diversity of study designs, outcome measures, and educational contexts, random-effects models were used throughout, with inverse-variance weighting to account for within- and between-study variability [41].

For studies employing pretest–posttest designs, repeated-measures meta-analyses were conducted to assess within-session conceptual gains. Interpreting repeated-measures meta-analyses based on pretest–posttest effect sizes warrants caution, as these designs are more prone to bias. Repeated-measures designs violate the independence assumption of meta-analysis, and observed intervention effects may be confounded with natural change, reducing reliability [42]. To mitigate bias, pretest-posttest correlations should be incorporated into the computations [42]. Because such correlations are rarely reported in education studies (none in the present meta-analysis), we imputed *r* = 0.30, 0.50, and 0.70 and ran three analyses, each applying a single r across all studies to represent weak, moderate, and strong within-subject correlations.

For comparative studies, independent-samples meta-analyses were performed to compare learning outcomes between intervention and control groups. Standardized mean differences (SMD; Hedges’ g) were calculated using a random-effects model with inverse-variance weights, giving greater weight to studies with larger sample sizes. Effect sizes were interpreted according to Cohen’s benchmarks: small (0.20–0.49), medium (0.50–0.79), and large (≥ 0.80) with 95% confidence intervals (CIs) [43]. Learner perceptions of PI were synthesized using a proportional meta-analysis (survey responses/total respondents). Proportions were transformed using the logit transformation and pooled using random-effects models with inverse-variance weighting [44, 45]. Results are reported as pooled proportions with 95% CIs and two-sided p-values.

Between-study heterogeneity was assessed using Cochran’s Q statistic and quantified with the I² statistic, interpreted as nominal when I^2^ < 25% and considerable when I^2^ > 75% [46, 47]. We calculated the 95% prediction interval (PI) to estimate the expected range of effects in future comparable studies [48]. Publication bias was assessed via visual inspection of the funnel plots and statistical testing using Egger’s regression intercept and Begg and Mazumdar’s rank correlation [49].

## Results

### Search Results

**Fig. 2:**
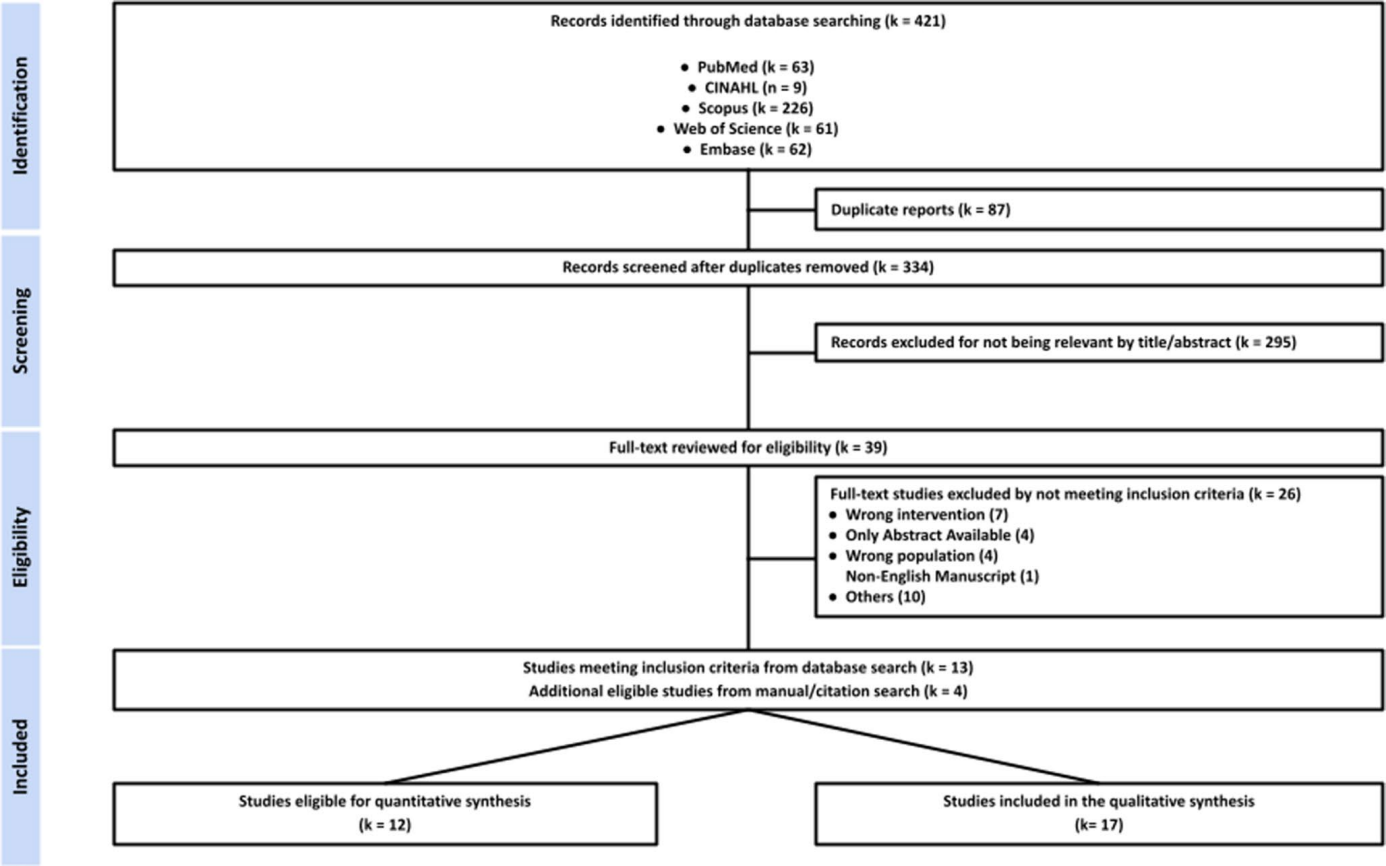
PRISMA flow diagram of the study selection process

The database search identified 421 records. After removing 87 duplicates, 334 records were screened at the title and abstract level. Of these, 39 articles were retrieved for full-text review. Thirteen studies met the inclusion criteria, and an additional 4 were identified through manual screening for a total of 17 included studies. Of these, 4 studies contributed to the repeated-measures meta-analysis (pretest–posttest conceptual gains), 4 studies to the independent-samples meta-analysis (PI versus control), and 7 studies to the proportional meta-analysis (learners’ perceptions of PI). The study selection process is summarized in the PRISMA flow diagram (Fig. 2).

### Study Characteristics

The 17 included studies were published between 2000 and 2025 and represented educational settings across nine countries. The United States accounted for the majority of studies (53%), followed by Japan, Denmark, and China (12% each), with single studies from Mexico and the Netherlands. The educational levels, totaling 2,166 learners, represented undergraduate medical education (UME; k = 13, *n* = 1712), graduate medical education (GME; k = 1, *n* = 53), combined UME and Graduate level (k = 2, *n* = 371), and undergraduate dental education (k = 1, *n* = 30). Sample sizes ranged from 30 to 336 participants, with a mean of approximately 145 students per study. Physiology was the most frequently investigated discipline (k = 7; 41%).

Methodologically, quasi-experimental designs were employed in 11 studies (65%), most commonly one-group pretest–posttest. Four studies (24%) were randomized controlled trials, and two (12%) used historical cohort comparisons. Studies integrated PI into curricula in two primary capacities: as a supplemental intervention added alongside an existing instructional format (k = 11) or as a direct replacement in which PI served as the primary mode of instruction (k = 5); one study employed both approaches. Intervention duration also varied substantially, from a single isolated instructional session (4 of 17; 24%) to serial implementations with multiple PI sessions (12 of 17; 71%), ranging from 2 to 45 sessions.

Overall, the included studies demonstrated substantial methodological heterogeneity across design, duration, and assessment tools, consistent with trends observed in contemporary meta-analyses of medical education. Table 1 summarizes the main study characteristics.

**Table 1:** Study characteristics (k = 17)

Author (Year)	Country / Institution	Education Level	Discipline / Topic	Study Design	Sample (*n*)	Primary Outcomes Reported	Preparation	Structure of Assessments	Intervention frequency	Instructional interventional approach ¹
Bian (2017) [50]	China / Kunming Medical University	UME	Physiology (lab)	Quasi-experiment (one group pretest-posttest)	135	Performance, confidence, perceptions, peer influence	Pre-session reading (1 week prior)	Identical pre–post (within-session, within-group)	Serial (across 6 sessions)	Replacement intervention
Bucheit (2015) [51]	USA / Mercer University	UME & GME	Interprofessional education	Quasi-experiment (one group pretest-posttest)	35	Perceptions	Pre-session questions (2 weeks prior)	Identical pre–post (within-session, within-group)	Isolated	Supplement intervention
Carpenter (2020) [52]	USA / McGovern Medical School	UME	Biochemistry	Quasi-experiment (one group pretest-posttest)	213	Perceptions, peer influence	Pre-session lecture record (time not specified)	Identical pre–post (within-session, within-group)	Not specified	N/A
Mitsuhashi (2020) [53]	Japan / Okayama University	UME	Epidemiology	Quasi-experiment (one group pretest-posttest)	121	Conceptual gain, peer influence	N/A	Identical pre–post (within-session, within-group)	Isolated	Supplemental intervention
Mitsuhashi (2021) [54]	Japan / Okayama University	UME	Epidemiology	Historical cohort	141	Affective domain	N/A	N/A	Isolated	Supplemental intervention
Mohammad (2021) [55]	Denmark / University of Copenhagen	UME	Respiratory physiology	Randomized controlled	186	Performance, perceptions	Pre-session reading (time not specified)	Identical pre–post (within-session, within-group), Identical post (post-intervention, between-groups)	Serial (across 3 sessions)	Supplemental intervention
Petersen (2014) [56]	Denmark / University of Copenhagen	UME	Acid–base physiology	Quasi-experiment (pretest-posttest with control group)	41	Performance, perceptions, peer influence	N/A	Identical pre–post (within-session, within-group), Identical pre–post (pre-post session, between-groups)	Serial (across 2 sessions)	Supplemental intervention
Rao (2000) [57]	USA / Wayne State University	UME	Respiratory physiology	Quasi-experiment (one group pretest-posttest)	256	Performance, peer influence	N/A	Identical pre–post (within-session, within-group)	Serial (across 10 sessions)	Supplemental intervention
Rao (2001)[58]	USA / Wayne State University	UME & Graduate Physiology	Respiratory physiology	Quasi-experiment (posttest only with control group)	336	Performance	N/A	Identical post (post-intervention, between-groups)	Serial (across 10 sessions)	Replacement intervention
Scalise (2025) [59]	USA / Univ. of Texas Dentistry	Undergraduate Dental Education	Dental anatomy	Randomized controlled	30	Performance, retention, perceptions	Pre-session quiz (1 week prior)	Identical pre–post (pre-post session, between-groups), Identical post (delayed post, between-groups)	Isolated	Supplemental intervention
Schuller (2015) [60]	USA / Northwestern University	GME	Surgical curriculum	Quasi-experiment (one group posttest), Historical Cohort	53	Engagement, retention, perceptions	Pre-session reading and questions (1 week prior)	Identical post (post-intervention, attendance-based within-group)	Serial (across 31 sessions)	Replacement intervention
Southwick (2007) [61]	USA / Univ. of Florida	UME	Infectious diseases / microbiology	N/A	N/A	Attendance	Pre-session questions (> 2 h prior)	N/A	Serial (across 1 academic year – sessions not specified)	Replacement intervention
Trout (2014)	USA / Wright State University	UME	Pharmacology	Quasi-experiment (one group posttest)	N/A	Performance	Pre-session lecture record and reading (time not specified)	Identical post (post-session, attendance-based within-group)	Serial (across 4 sessions)	Supplemental intervention
Vázquez-García (2018) [62]	Mexico / UNAM School of Medicine	UME	Human physiology	Quasi-experiment (one group pretest-posttest)	69	Performance, peer influence, retention	N/A	Identical pre–post (within-session, within-group), Identical post (post-intervention, within-group)	Serial (across 1 academic year – 45 sessions)	Supplemental intervention
Versteeg (2019) [63]	Netherlands / Leiden University Medical Center	UME	Cardiovascular physiology	Randomized controlled	317	Performance, transfer, peer influence	N/A	Identical pre (recall test, between-groups), Identical pre–post (within-session, between-groups)	Serial (across 2 sessions)	Supplemental intervention
Yelton (2017) [64]	USA / Wright State University	UME	Neuroscience, endocrinology, cardiology	Quasi-experiment (one group pretest-posttest)	92	Perceptions	Pre-session reading (time not specified)	N/A	Serial (across 1 academic year – sessions not specified)	Replacement and Supplemental intervention
Fu (2022) [65]	China / Chengde Medical University	UME	Human anatomy	Randomized controlled	141	Performance, perceptions	Pre-session reading (time not specified)	Identical post (post-intervention, between-groups)	Serial (not specified)	Supplemental intervention

¹ Replacement interventions were defined as those in which Peer Instruction (PI) replaced the existing instructional approach as the primary mode of instruction for the targeted curriculum (e.g., PI replacing a traditional lecturing system or conventional group work format). Supplemental interventions were defined as those in which PI was added to an existing non-PI instructional approach, or offered as an optional adjunct, without replacing the underlying instructional format (e.g., PI offered as an additional review session alongside traditional lectures).

### Quality of Included Studies

The total MERSQI scores across the 17 studies ranged from 5.5 to 13.5, with a mean score of 10.7 (SD = 2.2) and a median of 11. When considering only the 12 studies contributing to the meta-analyses, MERSQI scores ranged from 8 to 13.5, with a mean of 11.5 (SD = 1.5) and a median of 11.5. Overall, the quality of the evidence was moderate, with four studies achieving high scores ( > = 12.5) due to superior designs, specifically randomized controlled trials utilizing objective outcome measures. No studies were excluded based on a quality threshold.

### Repeated Measures Meta-analysis: Pretest versus Posttest Conceptual Gains

**Fig. 3:**
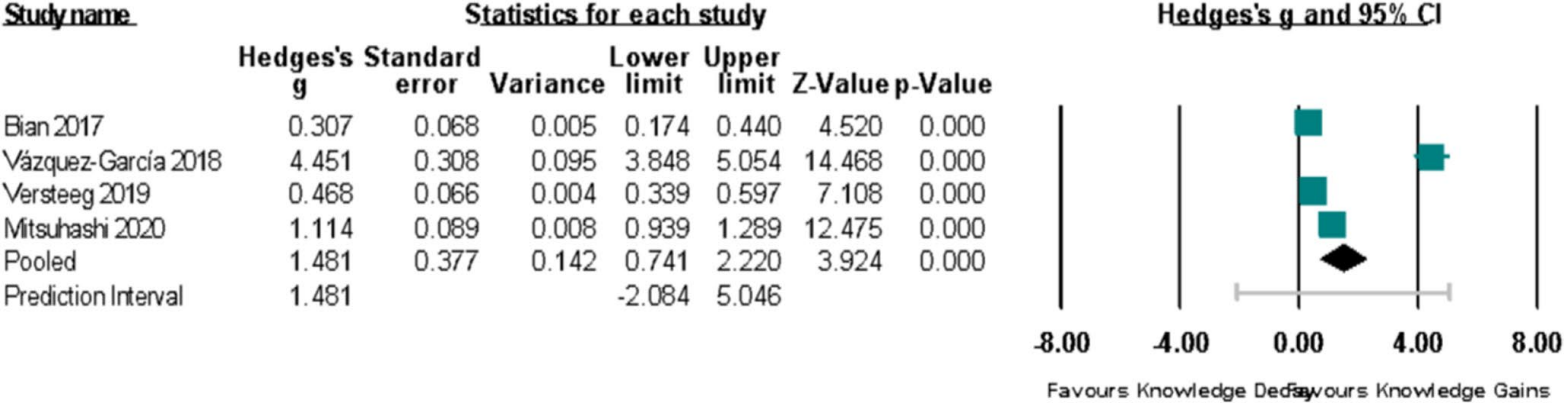
Forest plot of learner conceptual gains using an imputed pretest-posttest correlation of 0.70

**Fig. 4:**
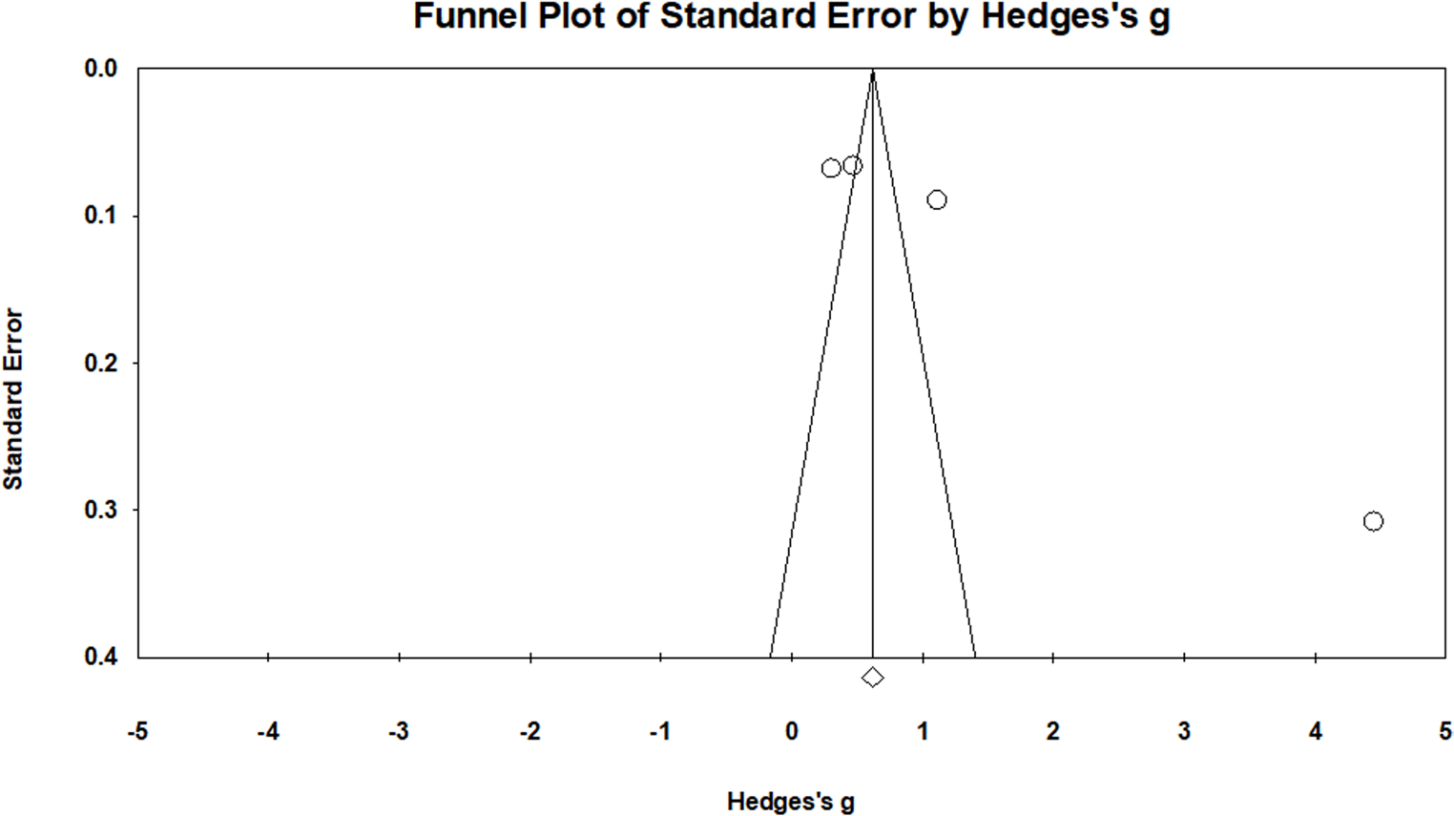
Funnel plot assessing publication bias in the repeated-measures meta-analysis of learner conceptual gains (Hedges’ g)

Across four studies with four interventions, students who participated in PI (*n* = 477) demonstrated statistically significant within-session conceptual gains, as indicated by a large positive summary effect size (SMD ≥ 1.481; *p* < 0.001; Table 2; Fig. 3). Heterogeneity across studies was high (Q ≥ 106.093; I2 ≥ 97%; *p* < 0.001; Table 2). Publication bias was probable, given that Egger’s test is generally more sensitive to asymmetry than Begg’s test, especially with few studies (Table 2; Fig. 4).

**Table 2:** Summary of pooled effect-size estimates (k = 4) arranged by pretest-posttest correlations

	Imputed Pretest-Posttest Correlations		
	0.30	0.50	0.70
Effect size (Hedges' g); p-value	1.525; <0.001*	1.521; <0.001*	1.481; <0.001*
95% Confidence interval	0.689 to 2.361	0.723 to 2.320	0.741 to 2.220
Q-statistic; *p*-value	106.093; <0.001*	140.560; <0.001*	212.294; <0.001*
I^2^ statistic	97%	98%	99%
Prediction interval	-2.439 to 5.489	-2.295 to 5.338	-2.084 to 5.046
Egger’s test *p*-value; Likelihood of publication bias	0.045*; Likely	0.043*; Likely	0.037*; Likely
Begg’s test *p*-value; Likelihood of publication bias	0.308; Unlikely	0.308; Unlikely	0.308; Unlikely

*Statistically significant at alpha = 0.05.

### Independent Samples Meta-analysis: Treatment versus Control

**Fig. 5:**
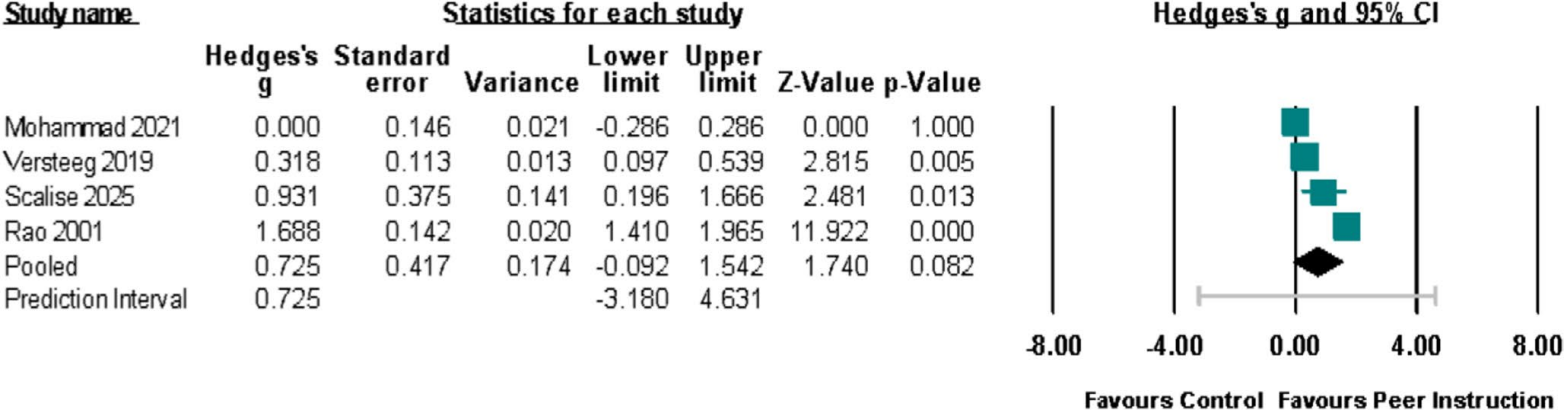
Forest plot of control versus peer instruction

A meta-analysis of 4 studies with 4 interventional comparisons, published between 2001 and 2025, revealed no effect (*p* = 0.082; Fig. 5) for the intervention when comparing PI (*n* = 512) against the control (*n* = 357). Heterogeneity across the included studies was high (Q = 82.730; I^2^ = 96%; *p* < 0.001); though, publication bias was unlikely (Egger’s Test *p* = 0.825, intercept = 2.37; Begg’s Test *p* = 1.000, Kendall’s Tau < 0.001). The limited number of comparative studies precluded moderator analyses. While conceptual gains were achieved, as demonstrated by the above pretest-posttest outcomes, these were generally comparable to those of the control group.

### Proportional Meta-analysis: Learners’ Perceptions

**Fig. 6:**
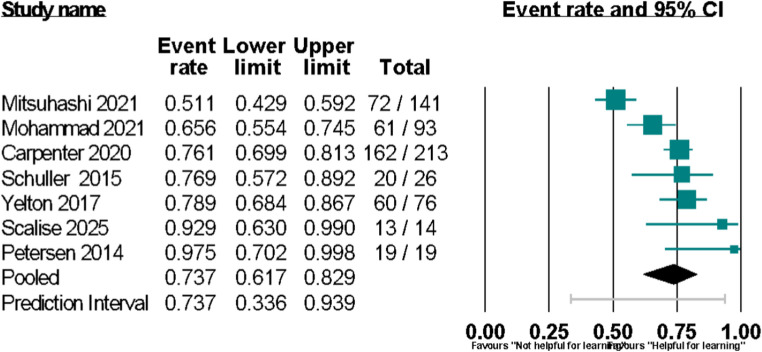
Forest plot of learners’ perceptions on the benefits of peer instruction

A proportional meta-analysis was conducted to summarize the extent to which PI was perceived to be beneficial to one’s learning. Across seven studies, 73.7% (CI at 95% = 61.7% to 82.9%; Fig. 6) of respondents perceived PI to have beneficial or helpful attributes (I^2^ = 84%, *p* < 0.001).

## Discussion

PI has been adopted inconsistently in medical and dental education, lagging behind its established implementation in other STEM disciplines. Related literature in these fields remains limited, with inconsistent terminology and frequent overlap between PI and broader peer-based or collaborative instructional approaches. The present systematic review and meta-analysis deliberately applies a narrow operational definition of Mazur’s PI, isolating it from adjacent pedagogical modalities. By synthesizing the available empirical evidence, the findings indicate that PI produces large within-session conceptual gains and is well received by learners but offers no clear performance advantage over other active approaches.

### Peer Instruction and Immediate Within-Session Conceptual Gains

A common finding across the literature is that PI is associated with significant within-session conceptual gains. Across four studies employing pretest-posttest designs, we found a large pooled effect size (Hedges’ g > = 1.48) favoring post-test scores over pre-test. This indicates that when learners engage with conceptual questions, discuss reasoning with peers, and receive structured instructor feedback, their understanding improves measurably over the course of the instructional encounter.

These findings align with well-established principles from cognitive and learning sciences, particularly social constructivism and Vygotsky’s Zone of Proximal Development (ZPD) (Vygotsky, 1978) [27]. PI uses peer-mediated explanations to facilitate learning within the ZPD, leveraging the cognitive proximity between learners. Explanations generated by peers who have recently mastered a concept may be more accessible than those offered by experts, whose reasoning has become more automated and abstracted [66, 67].

In addition, PI integrates several empirically supported learning mechanisms, including elaborative interrogation and immediate feedback [68, 69]. By requiring learners to commit to an answer and then defend it, PI induces epistemic curiosity and cognitive dissonance [70, 71]. The subsequent gain is likely the result of this dissonance being resolved through elaborative interrogation, which includes explaining why a fact is true or how it relates to prior knowledge. Furthermore, the “re-poll” phase of PI provides immediate feedback, a factor consistently associated with improved achievement across educational contexts [72]. These mechanisms may be particularly effective in cognitively demanding domains such as physiology, where understanding depends on mechanistic causal reasoning and dynamic systems thinking [73].

The large heterogeneity associated with these effects is also important. Some implementations appear more effective, while others produce only modest gains. This variability likely reflects differences in how PI was implemented: the quality of the questions posed, their alignment with learners’ prior knowledge, the time allotted for discussion, instructors’ facilitation skills, and the rigor with which the PI sequence is followed. In other words, PI is not a plug-and-play intervention. Its effectiveness depends heavily on its execution [22].

Finally, these observed within-session pretest–posttest gains are best understood as evidence that PI is instructionally efficient in medical and dental education, not that it transforms learners over time. The outcomes show that PI may help students conceptualize content during the session itself. Current empirical data is insufficient to support claims of superior long-term retention, cumulative course mastery, or downstream clinical competence. This is reflected in the present review, in which only three included studies examined learning beyond the immediate session, and their results did not consistently favor PI over other instructional approaches.

### Peer Instruction Does Not Demonstrate Clear Advantage over Alternative Instructional Methods

A comparative analysis of performance outcomes demonstrated that PI was non-superior to other pedagogical approaches (*p* = 0.082). This finding should be interpreted in the context of the comparator conditions employed. Notably, the control conditions in these studies were generally not passive lectures. In most cases, the controls involved some form of active or interactive learning. For example, one study used conventional laboratory instruction, another used audience response questions without peer discussion, another used collaborative activities with formative assessment, and another used self-explanation exercises. In essence, the comparisons were often “active learning vs. active learning” rather than “active vs. passive learning.” Thus, our results do not imply that PI offers no benefit over lecturing, and given the evidence from other fields, it likely would outperform a purely passive lecture if tested head-to-head [1, 3].

Under these conditions, the absence of between-group differences aligns with a growing body of evidence suggesting that multiple active instructional strategies can yield comparable learning outcomes when they engage similar cognitive processes [13]. This phenomenon reflects pedagogical equifinality, in which different instructional routes converge on identical cognitive end-states once a certain threshold of generative cognitive engagement is achieved [74, 75]. Within the ICAP framework, Constructive or Interactive learning activities produce superior gains over Passive or Active formats by requiring intensive knowledge-change processes, like integrating information into existing schemas and generating inferences beyond the provided material [69, 76]. Accordingly, when distinct instructional approaches impose similar levels of cognitive demand and learner engagement, variations in delivery format or sequencing are unlikely to produce meaningful learning gains [69, 75, 76]. Consequently, the decision to implement PI should be guided not only by expectations of superior exam performance, but also by alignment with the intended learning objectives, logistical feasibility, and faculty comfort.

Interpretation is further limited by marked heterogeneity across the comparative studies. PI varied considerably in its delivery. In addition, outcome assessments were administered at varying time points across studies, with posttests delivered immediately after the same session in some cases and after a sequence of sessions in others. These variations make it difficult to isolate the specific contribution of PI relative to the comparator. Without explicit passive learning controls and theoretical justifications for comparator selection, comparative studies can only determine whether one active learning approach marginally outperforms another.

In practical terms, our findings indicate that PI works as well as alternative instructional methods in medical education. For educators, this suggests that PI can be adopted without sacrificing educational effectiveness, but expectations of dramatic performance gains beyond those achieved by other active methods are not supported by current evidence.

### Learner Perceptions and Educational Acceptability

Across seven studies, approximately 74% of learners reported that PI supported their learning. The perception items evaluated PI in terms of helpfulness for learning, perceived academic improvement, support for diagnostic reasoning, or understanding of specific content areas. These findings align with prior work indicating that learners often perceive interactive instructional formats as more engaging and supportive than passive alternatives [17].

Positive learner reception has practical significance. In medical education, student buy-in influences attendance, engagement, and sustainability of instructional innovations. Some studies included in this review also reported improved class attendance and engagement when PI was implemented (e.g., Southwick 2007), further reflecting the high level of learner endorsement [61].

However, these findings should be interpreted with caution. A quarter of students in these studies were neutral or unconvinced about PI’s benefits, which reflects that PI is not uniformly experienced as advantageous. Moreover, positive learner experience, while relevant, does not establish instructional effectiveness nor resolve comparative pedagogical value. Students’ feelings that they learned may reflect increased engagement, social interaction, or perceived clarity rather than demonstrable learning gains [77]. Learners often equate the clarity of a peer’s explanation with their own mastery, a phenomenon known as the “illusion of competence [78].” As such, these positive perceptions should be viewed as evidence of feasibility and acceptability rather than as proxies for performance-based outcomes.

### Study Limitations

Although PI first appeared in the medical/dental education literature two decades ago, the number of studies meeting criteria for quantitative synthesis remains limited, particularly those employing comparative designs. Most studies relied on single-group or quasi-experimental designs. Differences in intervention duration, implementation protocols, and outcome measures introduce considerable heterogeneity, reducing the generalizability of pooled findings. In addition, the review focused primarily on preclinical, classroom-based medical education, with limited representation of graduate training or dental education. The findings should therefore be interpreted within this context and not generalized to clinical or workplace-based learning environments. As with all meta-analyses, this synthesis is subject to biases inherent in the underlying literature, including publication bias, research biases (e.g., sampling or interpretive bias), and dependence on empirical reports that may be selectively or incompletely reported [45].

According to a 2-tailed (alpha = 0.05) power analysis for meta-analysis (using the ‘metapower’ package in R Studio), in the presence of high heterogeneity (96%), four studies with an average of 89 participants per study and a summary effect size estimate of 0.725 yield a power of 0.361 for a random effect model. As such, the independent-samples meta-analysis was underpowered according to the conventional threshold of 0.80. We did not assess publication bias for the proportional meta-analysis because commonly used tests are not recommended for this use [79]. Moreover, as Barker et al. [79] note, the assumption that positive results are more likely to be published does not necessarily hold for proportional outcomes.

Lastly, the majority of included studies were in undergraduate medical courses (pre-clerkship science courses); only one study involved residents, and one involved dental students. Therefore, our conclusions are most applicable to these contexts in which PI is implemented as part of classroom-based instructional activities. Furthermore, these findings should not be extrapolated to other health professions (e.g., nursing and pharmacy, which we excluded), clinical teaching environments, or to more informal peer-teaching contexts that do not follow the structured PI format.

### Future Research and Directions

The evidence synthesized in this review indicates that research on PI is more developed within UME and has progressed beyond initial feasibility and proof-of-concept studies, while remaining limited in graduate medical and dental education. Although the available evidence suggests that PI is associated with meaningful learning gains across these settings, the literature is not yet sufficiently developed to support precise conclusions regarding its comparative educational value. Specifically, current studies do not yet delineate how, for whom, or under what conditions PI provides a distinct advantage over alternative instructional approaches. Advancing the literature will require a shift away from broad validation studies toward theory-driven, methodologically rigorous research. To date, many comparative studies have relied on loosely defined “control” conditions, limiting interpretation when between-group differences are absent. Comparative designs should instead be grounded in explicit learning theory, with careful articulation of which cognitive mechanisms are intentionally shared across conditions and which are experimentally isolated. Without this clarity, null findings remain ambiguous, as they may reflect overlapping underlying learning processes rather than true pedagogical equivalence.

A second priority concerns the durability of learning. While PI reliably produces short-term conceptual gains, little is known about whether these persist amid the rapid content turnover characteristic of preclinical curricula. Studies incorporating delayed post-tests are needed to determine whether PI meaningfully alters one’s knowledge decay or primarily enhances short-term performance. A related concern is transfer, as it remains uncertain whether the reasoning students develop through peer discussion translates into performance on novel clinical problems.

Third, heterogeneity in PI implementation must be examined more systematically. Treating PI as a uniform intervention obscures the influence of design features that likely moderate effectiveness, including question difficulty, alignment with learners’ prior knowledge, duration and structure of peer discussion, and the pedagogical approach to instructor feedback. Examination of these factors is necessary to identify the conditions under which PI is most effective.

Fourth, the included studies spanned nine countries with differing educational traditions and cultural norms. Variations in how students are expected to participate, engage in peer discussion, and interact with instructors may influence how PI is enacted and how learners respond to it. These contextual factors may influence PI effectiveness but could not be examined given the limited number of studies per country. Future studies should report institutional and cultural characteristics in sufficient detail to enable moderator analyses.

Finally, methodological advancement will require stronger study designs. The predominance of single-site, quasi-experimental studies limits generalizability and amplifies context-specific effects. Multicenter trials using shared protocols and standardized outcomes are essential for determining whether PI effects are robust across institutions and educational contexts.

## Conclusion

Mazur’s Peer Instruction format produces short-term gains in conceptual understanding in medical and dental education and is generally positively received by learners. When evaluated against alternative active instructional approaches, PI was noninferior in terms of learner performance outcomes. Implementation of PI varies widely across institutions. Evidence of PI’s longer-term knowledge retention and transferability to clinical and dental education contexts remains limited.

## Supplementary Information

Below is the link to the electronic supplementary material.

Supplementary Material 1
