# Supplementary material for: Mazur’s Peer Instruction in Medical Education: A Systematic Review and Meta-Analysis
Source: Med Sci Educ. 2026 Apr 28;36(3):1685–99. doi: 10.1007/s40670-026-02744-1 (PMC13356240; doi:10.1007/s40670-026-02744-1)
Supplement: Supplementary file 1 — Supplementary Material 1 [file 40670_2026_2744_MOESM1_ESM.pdf]

Appendix A. Database search strategy

Date of search: September 12, 2025

PubMed
(“Education, Medical, Undergraduate”[Mesh] OR medical-education[tiab] OR medical-student*[tiab] OR UME[tiab] OR undergraduate-medical-student*[tiab] OR “school of medicine”[tiab] OR MS1[tiab] OR MS2[tiab] OR MS3[tiab] OR MS4[tiab] OR "Education, Medical, Graduate"[Mesh] OR “graduate medical education”[tiab] OR "Education, Dental, Graduate"[Mesh] OR “school of dentistry”[tiab] OR dentistry-student*[tiab] OR dental-student*[tiab]) AND (peer-instruct*[tiab])

CINAH
(MM "Education, Medical+" OR MM "Students, Medical+" OR "medical student" OR "medical education" OR MS1 OR MS2 OR MS3 OR MS4 OR MM "Education, Dental") AND ("peer instruction")

Scopus
("medical student" OR "medical education" OR "medical school" OR "school of medicine" OR MS1 OR MS2 OR MS3 OR MS4 OR "dental education" OR "dental school" OR "school of dentistry") AND ("peer instruction") AND (LIMIT-TO (SUBJAREA , "MEDI"))

Web of Science
("medical student" OR "medical education" OR “medical school” OR “school of medicine” OR MS1 OR MS2 OR MS3 OR MS4 OR “dental education” OR “dental school” OR “school of dentistry”) AND ("peer instruction")

Embase
('medical education'/exp OR 'medical education' OR 'dental education'/exp OR ‘dental education’ OR ‘school of medicine’ OR ‘school of dentistry’) AND ('peer instruction')
